# Is minimally invasive esophagectomy effective for preventing anastomotic leakages after esophagectomy for cancer? A systematic review and meta-analysis

**DOI:** 10.1186/s12957-015-0661-z

**Published:** 2015-09-04

**Authors:** Can Zhou, Gang Ma, Xiao Li, Juan Li, Yu Yan, Peijun Liu, Jianjun He, Yu Ren

**Affiliations:** Department of Breast Surgery, the First Affiliated Hospital, Xi’an Jiaotong University, 277 Yanta Western Rd, Xi’an, 710061 Shaanxi Province China; Department of Translational Medicine Center, the First Affiliated Hospital, Xi’an Jiaotong University, Xi’an, 710061 Shaanxi Province China

**Keywords:** Minimally invasive esophagectomy, Open esophagectomy, Anastomotic leakages

## Abstract

**Background:**

Compared with open esophagectomy (OE), minimally invasive esophagectomy (MIE) proves to have clear benefits in reducing the risk of pulmonary complications for patients with resectable esophageal cancer. The objectives of our study were to explore the superiority of MIE in reducing the occurrence of anastomotic leakages (ALs) when compared to OE.

**Methods:**

A systematic review and meta-analysis was performed to assess the superiority of MIE on the occurrence of ALs over OE, by searching many sources (through December, 2014) such as Medline, Embase, Wiley Online Library, and Cochrane Library. Fixed-effects model was used to calculate summary odds ratios (ORs) to quantify associations between OE and MIE groups. Cochran’s Q and *I*^2^ statistics were used to evaluate heterogeneity among studies.

**Results:**

Among a total of 43 studies involving 5537 patients included in the meta-analysis, 2527 (45.6 %) cases underwent MIE and 3010 (54.4 %) cases underwent OE. Compared to patients undergoing OE, patients undergoing MIE did not have statistical significance in reduced occurrence of ALs (OR = 0.97, 95 % CI = 0.80–1.17). Insignificant reduced occurrence of ALs was not associated with anastomotic location (OR = 0.90, 95 % CI = 0.71–1.13) or anastomotic procedure (OR = 1.02, 95 % CI = 0.79–1.30).

**Conclusions:**

More proofs are needed to clarify the strengths or weaknesses of MIE in preventing anastomotic leakages after esophagectomy for cancer. A largely randomized, controlled trial should be undertaken to resolve this contentious issue urgently.

## Background

Esophagectomy is a primary curative modality for localized esophageal cancer. Esophagogastric anastomotic leakage (AL) is a devastating complication of esophageal resection and is associated with serious patient morbidity and mortality [1–3]. The reported incidence of ALs accounts for 40 % of postoperative fatalities after esophagectomy, with the frequency ranged from 4 to 17 % [2, 4, 5]. Although the etiology of ALs is multifactorial, anastomotic technical errors and occult ischemia of the mobilized gastric fundus are the two major causes [1, 6]. Thus, choosing what surgical technique is supposed to play a major role. For example, esophagogastric anastomoses after esophagectomy can be performed in the neck or chest, by a hand-sewn method or by using a mechanical stapling device [7, 8].

Minimally invasive esophagectomy (MIE), which was first described in the 1990s [9–11], was attributed to the superiority on a reduced risk of postoperative outcomes without compromising oncological outcomes, avoiding thoracotomy and laparotomy [12–17]. Therefore, theoretically, the procedure of MIE may have a significant impact on the reduction in ALs risk due to the introduction of long laparoscopic and thoracoscopic instruments. Nevertheless, after scrutinizing pertinent original research articles and randomized controlled trials, we found that this theoretical assumption has never been subjected to empirical verification. The main reason for this might be that previous studies focused too much on efficacy and safety rather than surgical techniques.

Therefore, at least two critical questions are of considerable interest and remain unanswered in esophageal surgery: (i) whether MIE has superiority in reducing the occurrence of ALs when compared to open esophagectomy (OE) and (ii) whether the anastomotic methods or sites of MIE have effect on prevention of ALs. For this, we conducted the present systematic review and meta-analysis to comprehensively explore the relation between ALs and MIE, as well as ALs and the concomitant anastomotic methods or sites, aiming to provide meaningful clues for future research and current clinical practice.

## Methods

### Literature search

Two independent observers searched the following databases in Medline, Embase, Wiley Online Library, and other sources such as the Cochrane Library from inception to December 15, 2014. The databases were searched using the terms minimally invasive esophagectomy, esophageal cancer, esophageal carcinoma, and open esophagectomy. This report complies with the preferred reporting items for systematic reviews and meta-analyses (PRISMA) [18].

### Study selection

Studies were selected for inclusion if they met the following criteria: (i) comparing MIE with OE, (ii) published in English or Chinese, (iii) randomized or non- randomized controlled study with parallel controls, and (iv) gray literatures such as conference proceedings, reports, and other peer-reviewed research.

The following studies were excluded: (i) interest was not reported or it was impossible to calculate the outcomes from the published results, (ii) not mentioning a distinct group of patients or comparing the outcomes of interest, and (iii) review articles, letters, comments, case reports, and unpublished articles (abstracts only).

### Quality assessment and data collection

The methodological quality of the included studies was independently assessed by two observers using the methodological index for non-randomized studies (MINORS) instrument, a quality assessment tool specifically developed for systematic reviews of non-randomized control studies [19]. The total quality score ranges from 0 (low quality) to 24 (high quality). The disagreements between the two observers were resolved by discussion with the corresponding author via e-mail or personal interview.

The information, extracted from each publication in the form of a table, included the following: authors, the nation of origin, the year of publication, the number and ages of the patients, etc. All eligible studies were retrieved and evaluated by two independent reviewers. Disagreements on inclusion were discussed with the guidance of the corresponding author via e-mail, if necessary.

### Outcomes definition

The definition of minimally invasive esophagectomy (MIE) is totally MIE, not including thoracoscopic/laparotomy assisted esophagectomy, or hybrid MIE. Anastomotic leakages, leak, and fistula uniformly referred to ALs.

### Statistical analysis

The primary outcome measure was ALs, as it was considered an important outcome indicator in esophageal surgery and had been used to compare outcomes among different medical institutions. Secondary outcome included the association of ALs and anastomotic sites or methods for patients under MIE.

After appropriate conversion, data from the various studies were combined using fixed-effects meta-analyses. Forest plots were provided with pooled odds ratios (ORs) and corresponding 95 % confidence intervals (CIs). Begg’s funnel plot was used to provide diagnosis of the potential publication bias [20]. Cochran’s chi-square-based *Q* statistic test was performed in order to assess possible heterogeneity between the individual studies and thus to ensure that each group of the studies was suitable for meta-analysis [21].Thresholds for interpretation of heterogeneity were adopted as outlined in the Cochrane Handbook: 0 to 40 %-low, 30 to 60 %-moderate, 50 to 90 %-possible substantial, and 75 to 100 %-considerable heterogeneity. If the heterogeneity was high [22] (*I*^2^ > 50 % or *P* < 0.10), a sensitivity analysis would be performed using the “metaninf” *Stata* command. If the heterogeneity was deemed to be considerable, we would not pool the results and provide a narrative assessment instead [23].All statistical processes were done in Stata version 12.0 (Stata Corp LP, College Station, TX, USA) software.

## Results

### Selected studies and methodological quality

**Fig. 1:**
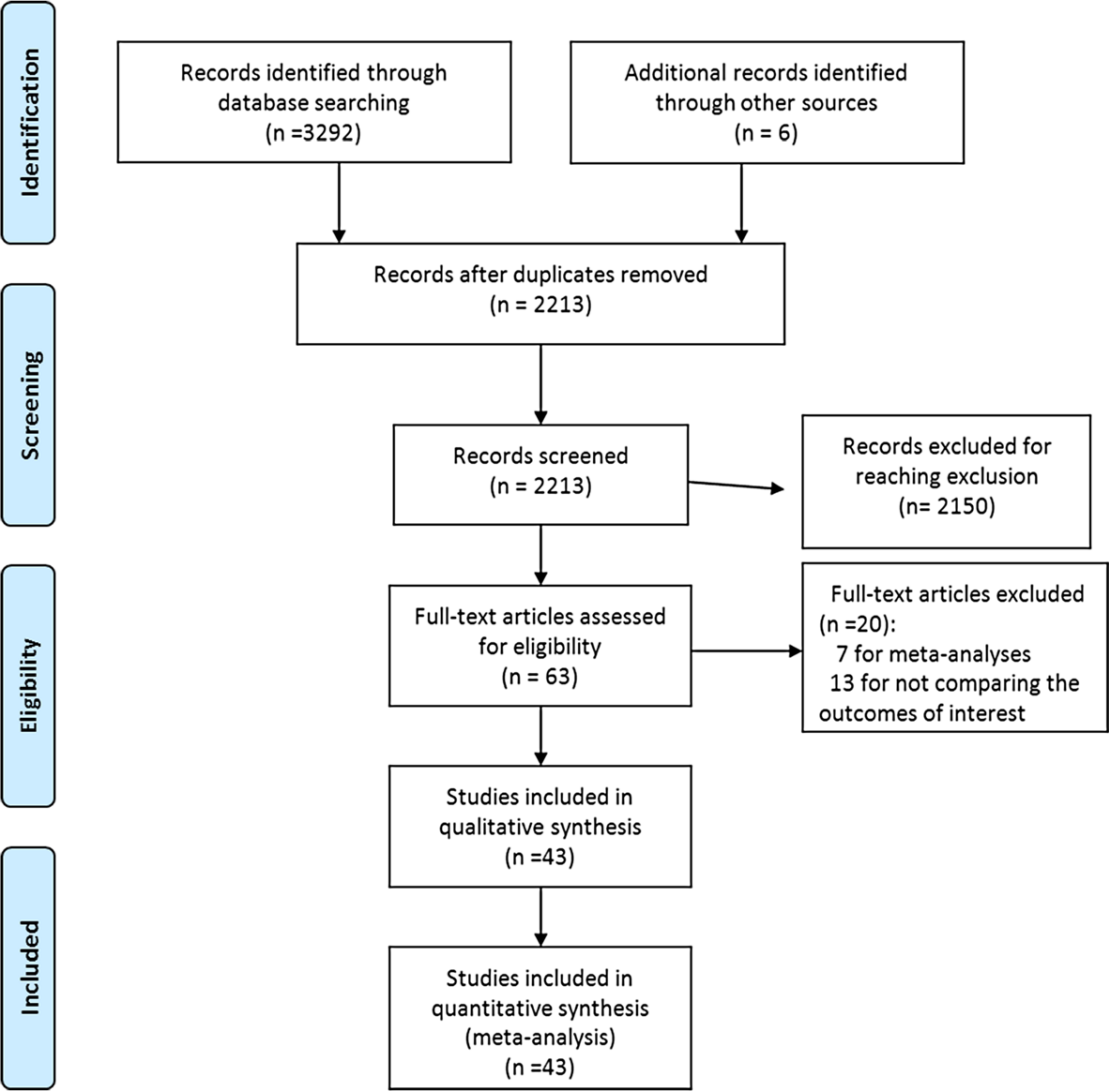
Flow diagram of the search and selection method

Figure 1 shows a flow diagram of our search and selection process. Forty-four studies were selected from the 57 studies, with the reason that 13 studies did not compare the outcomes of ALs. The evaluation results of the methodological quality of the studies are shown in Table 1. The total quality scores of the included studies ranged from 16 to 20 (Table 1). Randomized controlled design was done in only one study [13].

**Table 1:** Characteristics and demographics of included studies

Study	Year	Country	Design	Cases		ALs		MINORS	Adeno. %	Anastomosis	
				MIE	OE	MIE	OE			Location	Procedures
Smithers BM [42]	2007	Australia	P	23	114	1	10	20	70.6	Cervical	Hand-sewn
Kunisaki C [43]	2004	Japan	P	15	30	2	1	16	NA	Intrathoracic	Stapler
Law S [58]	1997	China	NA	18	63	0	2	16	NA	Cervical	Hand-sewn
Nguyen NT [24]	2000	USA	R	18	36	2	4	16	NA	Cervical	Stapler and hand-sewn
Kubo N [25]	2014	Japan	R	93	74	6	7	16	NA	Cervical	Unknown
Osugi H [44]	2003	Japan	P	72	77	1	2	20	0	Cervical	Unknown
Van den Broek WT [62]	2004	Netherlands	NA	19	14	2	3	19	71.1	Cervical	Hand-sewn
Braghetto I [26]	2005	Chile	R	47	60	3	10	20	NA	Cervical	Hand-sewn
Benzoni E [45]	2006	Italy	P	9	13	1	1	20	23.8	Cervical	Hand-sewn
Fabian T [27]	2006	USA	R	51	24	3	3	20	69.2	Cervical and intrathoracic	Unknown
Shiraishi T [28]	2006	Japan	R	78	37	9	9	16	NA	Cervical	Unknown
Bresadola V [64]	2007	Italy	NA	22	43	1	2	16	NA	Cervical	Hand-sewn
Perry KA [46]	2009	USA	P	21	21	4	6	16	45.2	Cervical	Unknown
Parameswaran R [60]	2009	UK	NA	50	30	4	1	19	82.5	Cervical	Stapler
Kitagawa H [54]	2009	Japan	NA	16	10	1	2	20	NA	Cervical	Hand-sewn
Zingg U [63]	2009	Australia	NA	56	98	11	11	20	72.1	Cervical	Hand-sewn
Saha AK [61]	2009	UK	NA	16	28	2	3	19	16+	Cervical	Stapler and hand-sewn
Hamouda AH [47]	2010	UK	P	24	51	3	0	16	80	Intrathoracic	Hand-sewn
Wang H [59]	2010	China	NA	27	29	5	4	16	5.3	Cervical	Unknown
Schoppmann SF [48]	2010	Australia	P	31	31	1	8	20	46.8	Cervical	Hand-sewn
Pham TH [14]	2010	USA	P	44	46	4	5	16	74.4	Cervical and intrathoracic	Stapler
Safranek PM [49]	2010	UK	P	41	46	7	1	16	NA	Cervical	Stapler and hand-sewn
Tsujimoto H [29]	2010	Japan	R	20	37	1	5	16	NA	Cervical and intrathoracic	Stapler and hand-sewn
Schröder W [30]	2010	Germany	R	238	181	18	17	16	60.1	Intrathoracic	Stapler
Berger AC [56]	2011	USA	NA	65	53	9	6	16	79.7	Cervical	Stapler
Yamasaki M [57]	2011	USA	NA	109	107	6	4	16	79.7	Cervical	Stapler
Gao Y [31]	2011	China	R	96	78	7	6	16	5.2	Cervical	Hand-sewn
Lee JM [50]	2011	Japan	P	30	64	2	18	20	5.1	Cervical	Stapler and hand-sewn
Nafteux P [32]	2011	Belgium	R	65	101	5	10	16	75.3	Cervical	Unknown
Sundaram A [33]	2012	USA	R	47	57	4	4	20	78.8	Cervical	Unknown
Sihag S [51]	2012	USA	P	38	76	0	2	16	85.1	Intrathoracic	Stapler
Maas KW [34]	2012	Netherlands	R	50	50	4	3	20	69	Cervical	Hand-sewn
Kinjo Y [52]	2012	Japan	P	106	79	8	10	19	3.2	Cervical	Unknown
Tsujimoto H [35]	2012	Japan	R	22	27	7	3			Cervical and intrathoracic	Stapler
Biere SS [15]	2013	Netherlands	RCT	59	56	7	4	18	61.7	Cervical and intrathoracic	Stapler
Noble F [53]	2013	UK	P	53	53	5	2	19	NA	Cervical and intrathoracic	Stapler
Ichikawa H [55]	2013	Japan	NA	153	162	14	27	20	66.7	Cervical and intrathoracic	Hand-sewn
Mu J [36]	2014	China	R	176	142	12	4	16	NA	Cervical	Stapler
Kauppi J [37]	2014	Finland	R	74	79	7	6	18	NA	Intrathoracic	Hand-sewn
Meng F [38]	2014	China	R	94	89	6	7	18	3.2	Cervical	Hand-sewn
Zhang J [39]	2014	China	R	60	61	3	2	16	NA	Intrathoracic	Stapler
Li J [40]	2014	China	R	89	318	19	45	20	2.2	Cervical and intrathoracic	Stapler and hand-sewn
Javidfar J [41]	2012	USA	R	92	165	5	7	18	91	Cervical	Unknown

### Characteristics of studies

The selected trials included a total of 43 studies and 5537 patients (Table 1). Among the included 43 studies, 19 were retrospective studies [15, 24–41], 13 were prospective ones [14, 42–53], and only 1 was randomized controlled trial (RCT) [13]. Ten studies were done in Japan [25, 28, 29, 35, 43, 44, 50, 52, 54, 55], 9 in USA [14, 24, 27, 33, 41, 46, 51, 56, 57], 7 in China [31, 36, 38–40, 58, 59], 5 in the UK [47, 49, 53, 60, 61], 3 in Netherlands [15, 34, 62], 3 in Australia [42, 48, 63], 2 in Italy [45, 64], and the remaining were conducted in Germany [30], Chile [26], Belgium [32], and Finland [37].

### Type of surgery

**Fig. 2:**
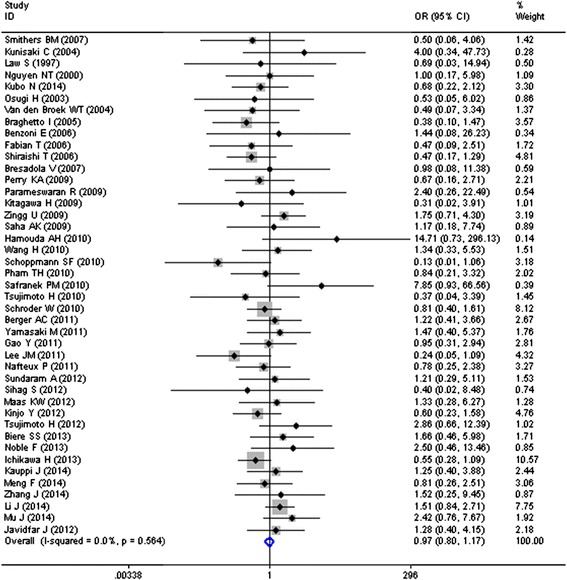
MIE and risk of anastomotic leakages (ALs)

Data for ALs were available for 43 studies totaling 5537 patients, of whom 2527 (45.6 %) patients underwent MIE and 3010 (54.4 %) underwent OE, with an overall ALs rate of 9.2 % (509/5537). The pooled OR of 0.97 (95 % CI = 0.80–1.17), as shown in Fig. 2, indicated no significant reduction in the risk of ALs after MIE when compared with OE, with no heterogeneity among results from different studies (*I*^2^ = 0.0 %, *P* = 0.564).

### Anastomotic location of MIE

**Fig. 3:**
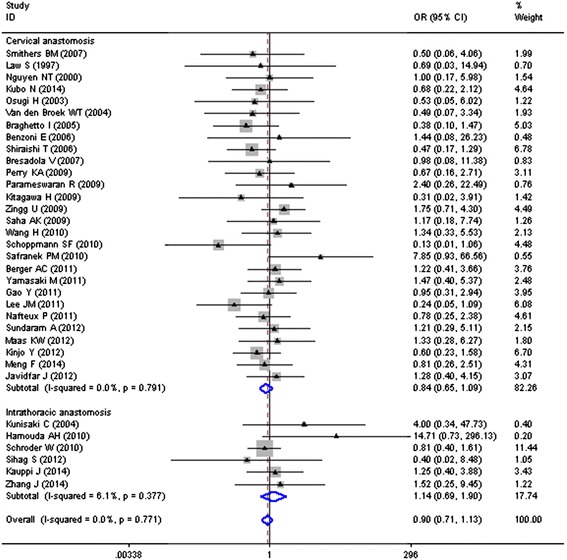
Anastomotic location of MIE and risk of anastomotic leakages (ALs)

The analysis on anastomotic location of MIE on ALs status was based on 34 trials or 4005 participants. An insignificant effect of the anastomotic location of MIE in ALs status (OR = 0.90, 95 % CI = 0.71–1.13) was showed in Fig. 3, with no statistical heterogeneity (*I*^2^ = 0.00, *P* = 0.771).

Twenty-eight studies, including a total of 3078 patients, had cervical anastomosis as anastomotic location for MIE, with an overall rate of 9.0 % (278/3078). The summary OR 0.84, as shown in Fig. 3, indicated that no significant reduction of 14 cases per 1000 individuals treated with MIE (95 % CI = 0.65–1.09) when compared with OE, with no heterogeneity among results from different studies (*I*^2^ = 0.0 %, *P* = 0.791).

The remaining 6 studies totaling 927 patients went through thoracic anastomosis as anastomotic location for MIE, with an overall ALs rate of 6.6 % (61/927). However, fix-effects pooled analysis (OR = 1.14, 95 % CI = 0.69–1.70) suggested that the incidence of ALs was not significantly higher when compared with OE (33/449, 7.3 vs. 5.8 %, 28/478), with no heterogeneity among results from different studies (*I*^2^ = 6.1 %, *P* = 0.377), as shown in Fig. 3.

### Anastomotic procedure of MIE

The analysis on anastomotic procedure of MIE on ALs status was based on 27 trials totaling in 3478 participants. No statistical significance effect of anastomotic procedure of MIE in ALs status (OR = 1.02, 95 % CI = 0.79–1.30) was showed in Fig. 3, with no statistical heterogeneity (*I*^2^ = 0.00, *P* = 0.539).

**Fig. 4:**
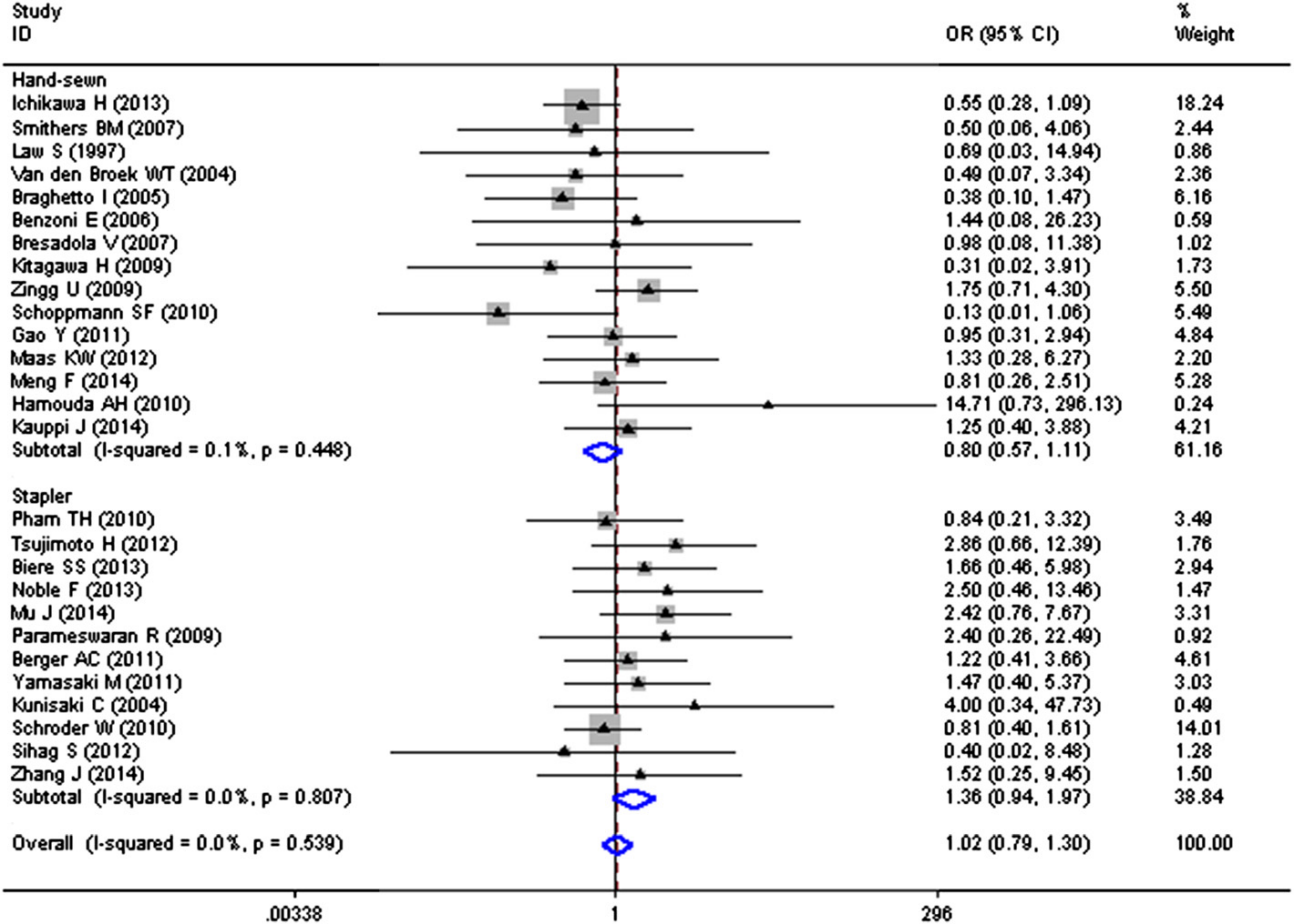
Anastomotic procedure of MIE and risk of anastomotic leakages (ALs)

Fifteen studies including 1687 cases had hand-sewn anastomosis as anastomotic procedure for MIE, with an overall rate of 9.5 % (160/1687). However, as shown in Fig. 4, the pooled OR was 0.80 (95 % CI = 0.57–1.11) which indicated insignificant reduction of 18 cases per 1000 individuals treated with MIE (62/732, 8.5 vs. 10.3 %, 98/955), with no heterogeneity among results from different studies (*I*^2^ = 0.1 %, *P* = 0.448).

Twelve studies and 1791 cases investigated stapler anastomosis as anastomotic method for MIE, with an overall ALs rate of 7.1 % (128/1791). The pooled OR was 1.36 (95 % CI = 0.94–1.97), showing insignificantly absolute decrease of 24 patients per 1000 individuals treated with MIE, with *no* heterogeneity (*I*^2^ = 0.0 %, *P* = 0.807), as shown in Fig. 4.

### Publication bias and sensitivity analysis

**Fig. 5:**
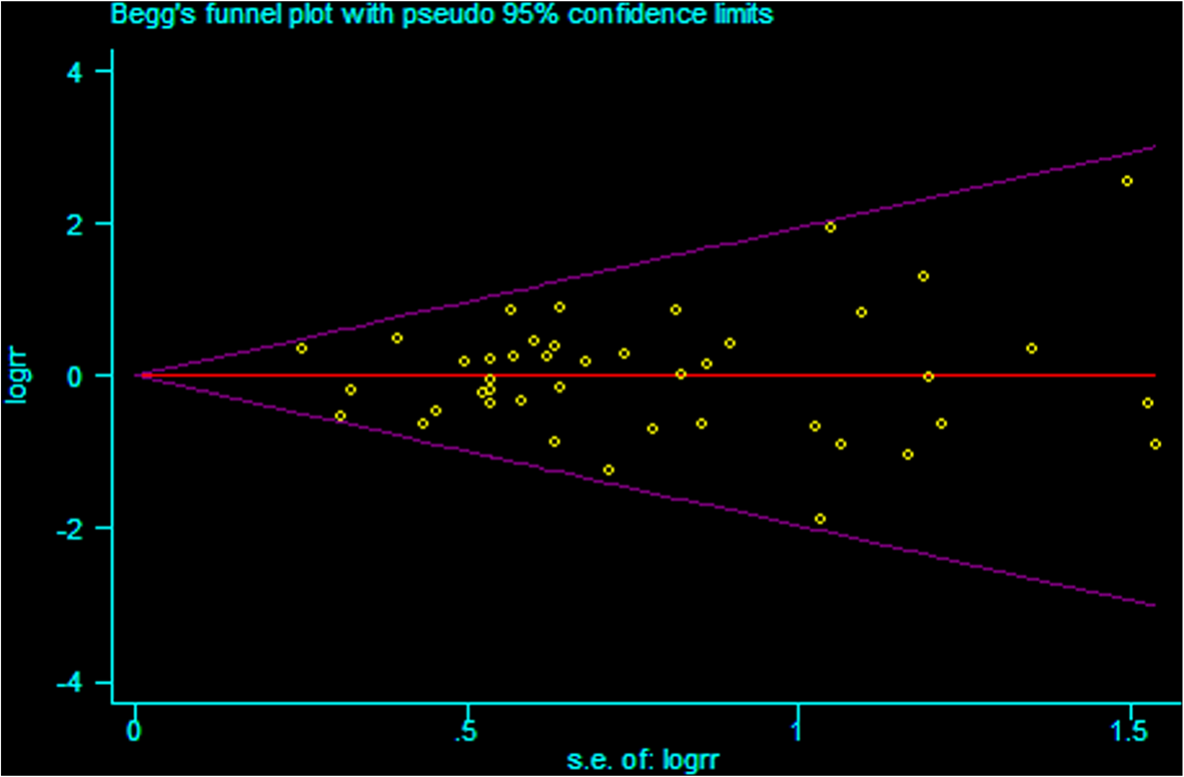
Begg plot of included studies

We plotted Begg’s funnel plot (Fig. 5) to examine small study effects. We also used Begg’s and Egger’s weighted regression method to calculate *P* values for bias. As shown in Fig. 5 the shape of the funnel plots did not reveal any evidence of obvious asymmetry. The *P* values of the Egger’s test were 0.869, 0.578, and 0.417 for type of surgery, anastomotic location of MIE, and anastomotic procedure of MIE, respectively, implying no existence of publication bias.

The influence of each study on the pooled ORs was examined by repeating the meta-analysis while sequentially omitting individual studies. Sensitivity analysis for type of surgery, anastomotic location of MIE, and anastomotic procedure of MIE indicated that no single study influenced the pooled ORs qualitatively, suggesting that the results of our meta-analysis are stable.

## Discussion

In our systematic review and meta-analysis, data to study the superiority of MIE on the occurrence of ALs was assessed in 43 studies totaling 5537 patients. We showed in a comprehensive systematic review that the use of stapler anastomosis for MIE might be insignificantly associated with the occurrence of ALs when compared with OE, irrespective of anastomotic locations or procedures.

As we described previously, resection for carcinoma of the esophagus with gastric transposition is currently considered as the standard surgical treatment for cure or palliation of esophageal cancer patients. Stomach is used most commonly for restoring gastrointestinal continuity through esophagogastrostomy anastomosis [7, 8]. AL after esophageal resection for cancer is supposed to be a severe complication, which contributes to considerable mortality and poor healing quality. In our analysis, the occurrence of ALs was 9.2 % (509/5537), which was consistent with the frequency 4–17 % reported in the previous papers. The prevention of ALs appears quite important in clinical research. Anastomotic technical errors and occult ischemia of the mobilized gastric fundus were regarded as the two major causes in the etiology of ALs. In comparison with OE technique, we also found the AL rate tended to reduce after cervical anastomosis but increase after intrathoracic anastomosis. The result was inconsistent with the previous report that MIE was associated with an increased incidence of AL due to the traumatic handling of the gastric conduit and the pressure transmitted through rigid laparoscopic instruments during mobilization, which resulted in increased tissue ischemia [65, 66]. The discrepancies could be attributed by the differences in the size and design of included studies.

The classical open approaches for esophageal resection include resection with a left thoracotomy, a thoracoabdominal resection approach and left neck, thoracoabdominal resection approach, also named as Sweet esophagectomy, Ivor Lewis esophagectomy, and McKeown-type esophagectomy. We know that esophagogastric anastomoses after esophagectomy can be performed in the neck for Sweet and Ivor Lewis approaches or the chest for three-incision approach [7, 8]. Moreover, whether the anastomosis should be performed in the neck or thorax is still a highly controversial issue in reconstruction after esophagectomy. Some authors favor anastomoses in the neck despite an increased incidence of leakage and damage to the recurrent laryngeal nerve due to better tumor eradication and reduced mortality and morbidity associated with an anastomotic breakdown [67, 68]. Others favor thoracic anastomosis for a lower but more ominous leakage rate due to less esophagi removed but decreased margins [69]. Consequently, anastomotic locations were assumed to have effect on leakage rates in the construction of intrathoracic and cervical esophagogastric anastomosis. In our analysis, we found the frequency of AL occurred in 8.3 and 9.7 % participators in MIE and OE groups, respectively, when performed with cervical esophagogastric anastomosis. However, no significant differences in the occurrence of ALs between the two groups were found when performed with thoracic or cervical esophagogastric anastomosis. Therefore, more proofs were needed to clarify the strengths and weaknesses of each anastomotic location.

We know that esophagogastric anastomoses can be performed via hand-sewn or stapled techniques by using mechanical stapling devices. Mechanical stapled anastomotic techniques, which were first described in 1977 [70], were deemed to have advantages of reduced time and likelihood of esophagogastric anastomotic failure, owing to the relatively ischemic gastric conduit resulting from staple distribution, staple closure, and the more uniform anastomotic tension along the entirety of the anastomosis [71]. Many reports showed that mechanical stapled anastomoses can decrease the rate of leakage after esophagogastrostomy [72, 73]. In our analysis, we failed to demonstrate a statistically significant difference in anastomotic leak rate after the comparison of MIE and OE undergoing hand-sewn or stapled technique. This consistent result further partly corroborates the existing evidence that mechanical stapled anastomoses can decrease the rate of leakage after esophagogastrostomy [72, 73]. The underlying reason may be the similarity in the adequately exposed operative field and level of ischemic gastric conduit. Nevertheless, our results indicated a tendency that the application of MIE could reduce the rate of ALs by hand-sewn anastomoses but increased the rate of ALs by stapled anastomoses. Therefore, there is insufficient evidence to clarify the strengths or weaknesses of MIE in preventing anastomotic leakages after esophagectomy for cancer.

Our meta-analysis has some limitations that might affect the interpretation of the results. First of all, among the included studies, only one was a RCT. The remaining 43 studies included were case-control or cross-sectional studies, which were susceptible to recall and selection biases. Therefore, to some degree, the studies included could not provide better evidence for potential treatment effects/harms than RCT. There might have been some underreporting of weight and overreporting of height, which might have led to an underestimation of the OR for this association. Secondly, there existed differences in study designs, demographics of participants, standardized protocols, histopathological types, and the characteristics of the tumor (poor tumor differentiation or advanced TNM stage). However, despite the use of appropriate meta-analytic techniques, we are unable to account for these differences, which may result in an overestimation or underestimation of the effect of MIO. Thirdly, unmeasured or residual confounding was likely to be present, such as preoperative nutritional status, intraoperative collateral tissue damage, bleeding, worsening organ failure due to surgical trauma, or difference of surgical techniques among the surgeons in included studies. Finally, we have to admit that patients selected for minimally invasive surgery are more likely to be in early stages of cancer, with smaller tumors and less risk of complications occurrence than patients in late stages.

## Conclusions

Minimally invasive esophagectomy seems to have a significant impact on the reduction in ALs risk due to the introduction of long laparoscopic and thoracoscopic instruments. A systematic review and meta-analysis can help to confirm the superiority.

Currently, there is no evidence to clarify the strengths or weaknesses of MIE in preventing anastomotic leakages after esophagectomy for cancer. A largely randomized, controlled trial should be undertaken to resolve this contentious issue urgently.
